# Depression Is Associated with the Aberration of Resting State Default Mode Network Functional Connectivity in Patients with Amyloid-Positive Mild Cognitive Impairment

**DOI:** 10.3390/brainsci13071111

**Published:** 2023-07-22

**Authors:** Sheng-Min Wang, Dong Woo Kang, Yoo Hyun Um, Sunghwan Kim, Chang Uk Lee, Hyun Kook Lim

**Affiliations:** 1 Department of Psychiatry, College of Medicine, The Catholic University of Korea, Seoul 06591, Republic of Korea; 2 Department of Psychiatry, Yeouido St. Mary’s Hospital, College of Medicine, The Catholic University of Korea, Seoul 07345, Republic of Korea; 3 Department of Psychiatry, Seoul St. Mary’s Hospital, College of Medicine, The Catholic University of Korea, Seoul 06591, Republic of Korea; 4 Department of Psychiatry, St. Vincent Hospital, Suwon, Korea, College of Medicine, The Catholic University of Korea, Suwon 16247, Republic of Korea

**Keywords:** depression, mild cognitive impairment, amyloid, Alzheimer’s disease, functional connectivity, default mode network

## Abstract

Mild cognitive impairment (MCI) is an intermediate stage between normal aging and dementia, and a significant number of individuals with MCI progress to develop dementia. Depression is prevalent in MCI patients and has been found to influence the disease progression of MCI. The default mode network (DMN), a brain network associated with Alzheimer’s disease (AD), and its functional connectivity might be a neurological mechanism linking depression and AD. However, the relationship between depression, DMN functional connectivity, and cerebral beta-amyloid (Aβ) pathology remains unclear. This study aimed to investigate DMN functional connectivity differences in Aβ-positive MCI patients with depression compared to those without depression. A total of 126 Aβ-positive MCI patients were included, with 66 having depression and 60 without depression. The results revealed increased functional connectivity in the anterior DMN in the depression group compared to the non-depression group. The functional connectivity of the anterior DMN positively correlated with depression severity but not with Aβ deposition. Our findings suggest that depression influences DMN functional connectivity in Aβ-positive MCI patients, and the depression-associated DMN functional connectivity aberrance might be an important neural mechanism linking depression, Aβ pathology, and disease progression in the trajectory of AD.

## 1. Introduction

Alzheimer’s disease (AD) is a neurodegenerative disorder characterized by progressive cognitive decline and memory impairment [1,2]. Mild cognitive impairment (MCI) represents a transitional stage between normal aging and dementia due to AD (AD dementia), and a substantial proportion of individuals with MCI eventually progress to develop AD dementia [3,4]. However, not all individuals with MCI exhibit the same clinical manifestations or disease trajectories [5,6].

One important factor that may influence the clinical presentation of MCI is the presence of depression, which has been found to be prevalent in patients with MCI [7]. AD is known to increase the risk of depression, but depression also increases the risk of subsequent AD [8]. More recent evidence suggests that depression is a causal factor accelerating MCI progression to AD dementia by facilitating beta-amyloid (Aβ) pathology [9]. However, the exact mechanisms underlying the association between depression and AD remain unclear.

Large-scale brain networks such as the default mode network (DMN) are known to be disrupted in patients with the trajectory of AD [10]. Emerging evidence suggests that alterations in functional connectivity within specific brain networks, such as the default mode network (DMN), may play a crucial role in linking depression and AD [11,12]. Studies showed that MCI patients with depression had lower resting state functional connectivity in the regions of DMN than in MCI patients without depression [13,14]. However, others showed that in MCI patients with depression functional connectivity belonging to DMN was either increased or not different when compared to MCI patients without depression [15,16,17].

The exact cause of these contradictory findings is still not clear. However, Aβ deposition is known to influence the association between DMN and numerous other AD-related measures. Studies showed that brain regions particularly vulnerable to early Aβ deposition overlap with brain regions of the DMN [18]. A longitudinal study showed that in patients with MCI when inter-network correlations between DMN and dorsal attention network were positive, a higher Aβ burden was associated with greater memory decline. In contrast, when the inter-network correlations were negative, there was no association between the magnitude of Aβ burden and memory decline [19]. Others showed that the lower DMN connectivity was associated with faster cortical thinning only in those with elevated baseline Aβ deposition [20]. A more recent study showed that the level of Aβ accumulation demonstrated differential effects on the functional connectivity of DMN [21]. For those having high Aβ accumulation, the Aβ deposition was associated with a reduction in DMN functional connectivity. For those having low Aβ deposition, the association was reversed and showed a positive correlation between Aβ deposition with DMN functional connectivity. 

Taken together, contradictory results regarding the depression-associated DMN functional connectivity changes might be due to additional neurobiological mechanisms cerebral Aβ pathology play in between depression and MCI [22]. Studies showed that cerebral Aβ retention was associated with the aberrance of DMN functional connectivity even in the absence of depression or cognitive dysfunction [23,24]. In the other perspective, patients with depression showed increased functional connectivity in the anterior DMN in association with depressive symptoms and decreased functional connectivity in the posterior medial DMN in association with episodic memory decrement, and these functional connectivity aberrations occurred independent of Aβ pathology [25,26]. We previously showed that Aβ-associated DMN functional connective disturbances could be more pronounced in patients with depression than in people without depression, and the effects of cerebral Aβ retention on the severity of depression were mediated by DMN functional connectivity [22].

Considering all these factors, both depression and cerebral Aβ retention can have an influence on DMN functional connectivity in the trajectory of AD [22,23,24,27]. However, no previous studies investigated the effect of depression on the aberrance of DMN functional connectivity in MCI patients with cerebral Aβ deposition. Thus, previous studies failed to elucidate whether DMN functional connectivity in MCI patients with Aβ pathology is altered in response to depression per se or is aberrated due to Aβ pathology. In addition, Aβ-positive MCI patients with depression are known to have a higher risk of developing AD dementia than Aβ-positive MCI patients without depression [28], but the underlying neurobiological mechanisms are poorly understood. 

Understanding the multifaceted relationships between depression and DMN functional connectivity in MCI patients with cerebral Aβ deposition could provide valuable insights into the development and progression of AD and inform potential targets for therapeutic interventions. To fill in this gap, we aimed to investigate the DMN functional connectivity differences between patients with depression and those without depression, both of whom have MCI and are positive for Aβ deposition as confirmed by amyloid positron emission tomography (PET) imaging. We hypothesized that Aβ-positive MCI patients with depression will exhibit altered DMN connectivity compared to Aβ-positive MCI patients without depression.

## 2. Materials and Methods

### 2.1. Participants 

This is a single-center, cross-sectional study which used data from Catholic Aging Brain Imaging (CABI) database. The CABI database contains brain scans of patients who visited the outpatient clinic at Catholic Brain Health Center, Yeouido St. Mary’s Hospital, The Catholic University of Korea, between 2017 and 2022. The inclusion criteria for all subjects were: (1) age ≥ 60 years and (2) confirmed to have cerebral Aβ deposition in amyloid-PET scan using ^18^F-flutemetamol (^18^F-FMM) (3) having magnetic resonance images (MRI) scans within 6 months before or after the amyloid-PET scan (4) received neuropsychological test with the Korean version of the Consortium to Establish a Registry for Alzheimer’s Disease (CERAD-K), which includes a verbal fluency (VF) test, the 15-item Boston Naming Test (BNT), the Korean version of the MMSE, word list memory (WLM), word list recall (WLR), word list recognition (WLRc), constructional praxis (CP), and constructional recall (CR) [29]. All subjects met the criteria for MCI as follows: (1) the presence of memory complaints corroborated by an informant; (2) objective cognitive impairment in more than one cognitive domain on CERAD-K (at least 1.0 standard deviation (SD) below age- and education-adjusted norms), (3) intact activities of daily living (ADL); (4) CDR of 0.5; (5) not demented according to the Diagnostic and Statistical Manual of Mental Disorders (DSM)-V criteria. The no-depression group had no clinically significant psychiatric disorders (depressive disorder, schizophrenia, or bipolar disorder). The depression group had additional inclusion criteria as follows: diagnosis of major depressive disorder according to the Diagnostic and Statistical Manual of Mental Disorders (DSM)-V criteria [30] with 17-item Hamilton Depression Rating Scale (HAMD_17_) total score > 14 [31].

We excluded subjects with the followings: (1) systemic diseases that can cause cognitive impairment, such as thyroid dysfunction, severe anemia, and syphilis infection; (2) severe hearing or visual impairment; (3) other neurological diseases that can cause cognitive impairment, such as clinically significant cerebrovascular diseases or traumatic brain diseases, cerebral hemorrhage, infarction, hydrocephalus, and epilepsy; (4) prescription medications that may cause changes in cognitive function; (5) contraindications for MRI examination or PET scan. Two geriatric psychiatrists, S.M.W and H.K.L, were responsible for the screening and diagnostic procedures. The study was conducted in accordance with the ethical and safety guidelines set forth by the Institutional Review Board of Yeouido St. Mary’s Hospital, The Catholic University of Korea (IRB number: SC20RISI0198). The informed consent was waived by the IRB because we only used retrospective data.

### 2.2. Acquisition of MRI

MRIs were acquired using a 3T Siemens MAGETOM Skyra machine and 32-channel Siemens head coils (Siemens Medical Solutions, Erlangen, Germany). A structural scan was acquired using a T1-weighted, magnetization-prepared, and rapid gradient-echo (MPRAGE) sequence with generalized, auto-calibrating, partially parallel acquisition (TE = 2.6 ms, TR = 1940 ms, inversion time = 979 ms, FOV = 230 mm, matrix = 256 × 256, and voxel size = 1.0 × 1.0 × 1.0 mm^3^). The resting state functional MRIs (fMRIs) were collected using a T2-weighted gradient echo sequence with TR = 2000 ms, TE = 30 ms, matrix = 128 × 128 × 29, and voxel size = 1 × 1 × 2 mm^3^. A total of 150 volumes were acquired over 5 min while patients were instructed to keep their eyes closed and think of nothing in particular. The MRIs were visually inspected for structural abnormalities and obvious artifacts due to head motion or dental materials.

### 2.3. Positron Emission Tomography 

We only included patients who were positive in ^18^F-FMM amyloid-PET scan. Information regarding ^18^F-FMM production, data collection, and analytical results were described previously [32]. The analysis of ^18^F-FMM-PET data was based on the standardized uptake value ratio (SUVR) 90 min post-injection. In terms of regional SUVR values, we measured six cortical regions of interest (frontal, superior parietal, lateral temporal, striatum, anterior cingulate cortex, and posterior cingulate cortex/precuneus) using PMOD Neuro Tool. The SUVR values of these six cortical ROIs were averaged to acquire the global Aβ burden or global SUVR values. Consistent with cutoff values used in previous ^18^F-FMM-PET studies, we used neocortical SUVR of 0.62 as the cutoff between high and low [32]. However, amyloid positivity was confirmed by visual readings from two separate nuclear medicine radiologists.

### 2.4. Data Analysis

#### 2.4.1. fMRI Data Preprocessing

We used the Functional Connectivity Toolbox (CONN, 18b), which is a MATLAB-based software that computes and analyzes functional connectivity using fMRI data to analyze resting-state functional connectivity. The fMRI data were preprocessed using the SPM12 pipeline in the CONN toolbox. The data were acquired in interleaved order, and slice-timing was corrected. To correct motion artifacts, we used the first image of the time series with a realignment procedure. For structural segmentation, SPM 12 pipeline was utilized to segment grey matter, white matter, and cerebral spinal fluids. Structural and functional images were normalized to the Montreal Neurological Institute (MNI) template. The pipeline also included functional outlier detection and scrubbing as well as functional spatial smoothing with a 6 mm Gaussian kernel. An anatomical component-based noise correction (aCompCor) procedure was used to remove possible confounders, including blood-oxygen-level-dependent (BOLD) signals from the white matter and CSF, realignment parameters (six motion parameters and six first-order temporal derivatives), scrubbing parameters (maximum inter-scan movement and identified invalid scans), and task-design effects. For the denoising step, the waveform of each brain voxel was filtered using a bandpass filter (0.009 < f < 0.08 Hz) to reduce the effects of low-frequency drift when removing white matter, CSF noise components, unwanted subject motion, and physiological noises.

In terms of region of interest-to-region of interest analysis, we carried out seed-to-voxel analysis with the posterior cingulate cortex (PCC), with coordinates 1, -61, 38, which is one of the major hubs of the DMN, as our seed [33]. The CONN toolbox provides 4 regions which can be utilized as seeds for the DMN: PCC, medial pre-frontal cortex, left parietal cortex, and right parietal cortex. Among them, PCC has been used most commonly as the seed when defining DMN due to multiple reasons. One of the first studies demonstrating the DMN with resting-state fMRI used the PCC as the seed because it had the highest peak z scores for task-related hypoactivation [34]. The PCC has been commonly used as the seed when investigating DMN in patients with depression [35]. Moreover, PCC extraction yielded the best results in discriminating patients of early MCI and late MCI [36]. Thus, we chose PCC as our seed. 

We first conducted first-level analyses involving the computation of seed-to-voxel connectivity maps for each subject. Thereafter, we used between-group differences controlling for sex and age to assess whether there were statistically significant differences in DMN among the depression and no depression groups. All the comparisons throughout the whole brain adopted voxel-wise statistics thresholded at *p* < 0.05, false discovery rate (FDR) corrected at cluster level, to resolve the problem of multiple comparisons.

#### 2.4.2. Morphometric Analysis

We used FreeSurfer software (version 6.0.0), Laboratory for Computational Neuroimaging at the Athinoula A. Martinos Center for Biomedical Imaging, Boston, MA, USA, which is available online at https://surfer.nmr.mgh.harvard.edu (accessed on 1 March 2021) to perform cortical reconstruction and volumetric segmentation of the entire brain [37]. The technical details have been described in previous publications [38], which included include removal of non-brain tissue using a hybrid watershed algorithm, bias field correction, automated Talairach transformation, and segmentation of subcortical white matter and deep gray matter structures. Intensity normalization and inflation of the cortical surface were then conducted to locate both the pial surface and the gray matter and white matter boundary. We used the shortest distance between the pial surface and the gray matter, and white matter boundary at each point across the cortical mantle was used to compute the cortical thickness [39]. The cortical map of each subject was smoothed with a Gaussian kernel of 10 mm full width at half-maximum (FWHM) for the entire cortex analyses. Lastly, we parcellated the cerebral cortex based on gyral and sulcal information implemented in FreeSurfer.

#### 2.4.3. Statistical Analysis

We used the free and open-source data analysis tool Jamovi (version 2.3.18.0), available online: https://www.jamovi.org (accessed on 1 April 2023), for statistical analysis of demographic data [40]. The two-sample independent t test was used to assess potential differences between the depression group and no depression group for all continuous demographic variables and clinical values. The Chi-square test was used for analysis of categorical variables. In all analyses, a two-tailed α level of 0.05 was taken to indicate statistical significance.

## 3. Results

### 3.1. Baseline Demographic and Clinical Data

A total of 126 Aβ-PET positive MCI patients were included in the study. The mean age was 78.02 (±6.76) and the mean global SUVR value was 0.757 (+0.091) which was sufficiently higher than cutoff value of 0.62. Among them, 66 subjects had depression (Aβ-positive MCI with depression = depression group) while 60 patients did not have depression (Aβ-positive MCI without depression = no depression group). The two groups did not show significant differences in age, sex, education, APOE E4 carriers, neuropsychological measures using CERAD-K, and global SUVR values. The depression group had significantly higher HAMD_17_ total scores than the no-depression group (Table 1).

### 3.2. Group Difference in Cortical Thickness and Functional Connectivity

**Figure 1:**
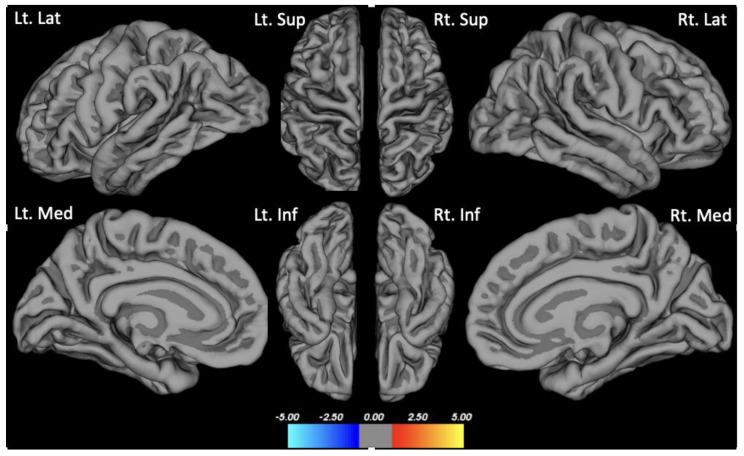
Group difference in cortical thickness. Clockwise from the upper left of the first row: Lt. Lat: Left lateral view; Lt. Sup: Left superior view; Rt. Sup: Right superior view; Rt. Lat: Right lateral view; Lt. Med: Left medial view; Lt. Inf: Left inferior view; Rt. Inf: Right inferior view; Rt. Med: Right medial view.

**Figure 2:**
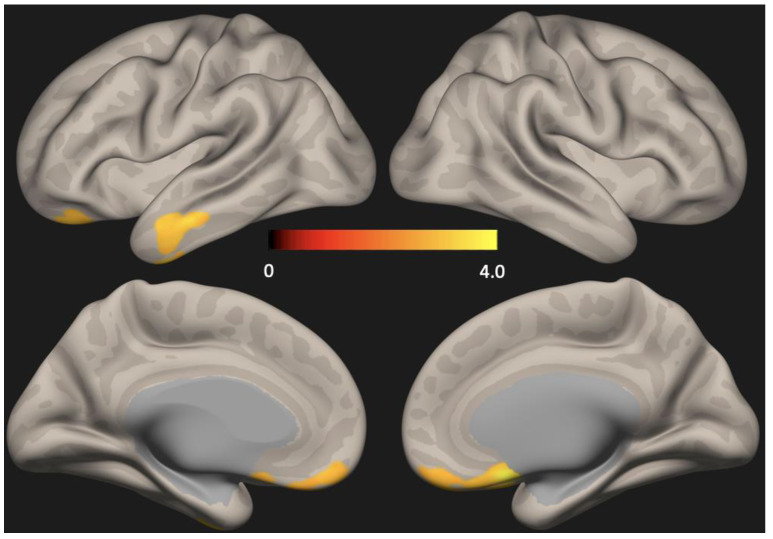
Statistical map representing group difference in default mode network (DMN) determined across all subjects. Seed-based analysis (PCC as the seed) showed increased functional connectivity in the anterior DMN (lateral temporal cortex and medial pre-frontal cortex) in the depression group compared to the no-depression group (*p* < 0.01, FDR corrected) PCC = Posterior cingulate cortex, FDR = False discovery rate.

There were no group differences in cortical thickness analyzed using Freesurfer (Figure 1). Seed-based analysis (PCC as the seed) showed increased functional connectivity in the anterior DMN (left lateral temporal cortex and medial pre-frontal cortex) in the depression group compared to the non-depression group (*p* < 0.01, FDR corrected) (Figure 2). 

**Figure 3:**
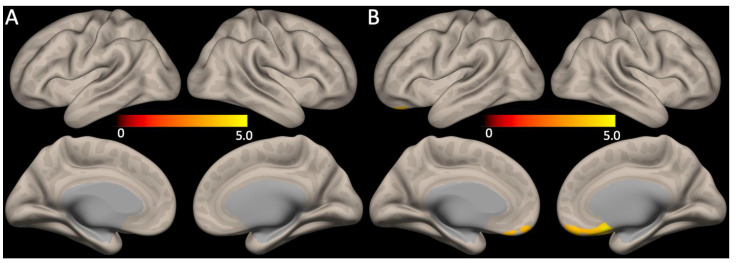
Association between DMN functional connectivity with Aβ retention or depression severity. (**A**) The global mean SUVR scores and the DMN functional connectivity did not show any correlations, whereas (**B**) functional connectivity of the anterior DMN (medial pre-frontal cortex) had positive correlations with the HAMD_17_ total scores (for all, FDR corrected *p* < 0.05).

No significant correlations between the global mean SUVR scores and functional connectivity were observed. However, the HAMD_17_ total scores showed a positive correlation with the medial pre-frontal cortex of the anterior DMN functional connectivity (Figure 3, FDR corrected *p* < 0.05).

## 4. Discussion

To the best of our knowledge, this is the first study investigating the effect of depression on DMN functional connectivity, one of the most important large-scale intrinsic networks associated with AD, in MCI patients having cerebral Aβ deposition. 

Altered functional connectivity of the DMN is known to be more prominent in patients with MCI when they present with cerebral Aβ deposition [41]. In addition, we previously showed that patients with depression have aberrance of DMN functional connectivity when compared with those not having depression [22]. In this study, we extended previous research by showing that (i) cortical thickness was not different but (ii) functional connectivity of the anterior DMN (lateral temporal cortex and medial pre-frontal cortex) was higher in Aβ-positive MCI with depression (depression group) than in Aβ-positive MCI without depression (no depression group). Studies suggested that failure to downregulate the anterior DMN activity in the resting state could be an important hallmark of depression [42]. Patients having MCI with depression are known to show subtle cognitive dysfunction when compared to MCI without depression [43]. Thus, the functional connectivity of the anterior DMN could have been increased as a compensatory response to the cognitive dysfunctions. In the other perspective, depressive symptoms might have occurred first and consequently exacerbated the inflammatory cascade in the brain area having Aβ deposition, which is the DMN [44]. The heightened neuroinflammatory process might have resulted in the increased DMN functional connectivity [45].

We further showed that in patients with Aβ-positive MCI (i) the DMN functional connectivity did not have significant associations with cerebral Aβ deposition severity or the global mean SUVR scores, but (ii) showed a positive correlation with the depression severity or the HAMD_17_ total scores. The DMN is known to be divided into two subparts; an anterior subdivision centered on the medial pre-frontal cortex and a posterior subdivision centered on the PCC and the precuneus cortex [46]. In line with our results, previous research showed that the anterior DMN is involved in the modulation of emotional behavior and self-referential processing, whereas the posterior DMN is mainly involved in the episodic memory [47]. Thus, our findings suggest that the anterior DMN functional connectivity in the depressed group might have increased in association with a heightened emotional response. In addition, Aβ-positive MCI patients having depression are at more than twice the risk of developing AD dementia than Aβ-positive MCI patients not having depression [28]. Higher neural activity is known to facilitate a detrimental cascade of the Aβ pathology [48,49], so the higher DMN functional connectivity associated with depression in Aβ-positive MCI might be an important neural mechanism linking depression, Aβ pathology, and disease progression from MCI to AD dementia. However, longitudinal studies are needed to confirm this hypothesis.

In contrast with previous studies, we did not find differences in functional connectivity in the posterior DMN between the depression and the no-depression groups [22]. The depression group and the depression group had comparable neuropsychological measures, with minimal differences in the memory domains. Thus, the episodic memory associated posterior DMN connectivity decrement in the depression group compared with the no-depression group might not have been prominent in our study. Contrary to previous studies which included both Aβ-positive and Aβ-negative MCI patients, we only included Aβ-positive MCI patients and investigated the effect of depression in the DMN connectivity [13,14,15,16,17]. Having equal Aβ burden or after removing the cofactor of varying Aβ deposition, our results might suggest that the increment of anterior DMN connectivity might be more closely related to the effect of the depression. However, further replication studies with MCI patients having diverse Aβ deposition levels and depression severity are needed to clarify this point. 

Multiple past studies showed that patients having depression are associated with lower cortical thickness or higher cortical atrophy than those not having depression [50,51]. Interestingly, the depression group and no depression in our study did not show a difference in cortical thickness analysis. Once again, having a homogenous group of patients with comparable Aβ deposition might be the cause because both Aβ pathology and depression are associated with cortical atrophy [52]. Moreover, the two groups in our study also had similar age, sex ratio, APOE E4 carriers, and neuropsychological profiles, which all are known factors influencing the degree of neurodegeneration. Regardless, our results might indicate that the depression associated with anterior DMN functional connectivity increment could occur in the absence of differences in Aβ deposition, cortical thickness, and cognitive dysfunction in patients with MCI. 

The present study had some strengths. We included subjects only with age ≥ 60 years and with MCI confirmed using the CERAD-K. Our strict selection criteria enabled us to include patients with balanced baseline demographic data and to prevent diverse confounding factors. In addition, by including MCI subjects having cerebral Aβ deposition only, we were able to investigate DMN functional connectivity changes associated with depression more clearly. In contrast, the study also has several limitations. This was a cross-sectional study, so we could only report associations and have limited ability to infer causal pathways. Further longitudinal analyses are needed to clarify causal relationships among depression, cerebral Aβ deposition, cognitive decline, and aberrance of DMN functional connectivity in the trajectory of AD. Second, we did not include MCI patients without cerebral Aβ deposition. Future studies comparing the DMN functional connectivity changes in response to both Aβ and depression (i.e., MCI with or without Aβ deposition and with or without depression) may further elucidate distinct and combined effects of depression and Aβ deposition in the DMN functional connectivity in patients with trajectory of AD. Lastly, a recent study utilizing both resting state fMRI and diffusion tensor imaging showed that the structural but not functional connectivity differences within DMN indicated conversion from MCI to dementia. However, we were not able to investigate the structural connectivity differences between groups because we did not undertake diffusion tensor imaging. Studies investigating the association among functional and structural connectivity, cerebral AB deposition, and depression are needed to clarify this point [53]. 

## Figures and Tables

**Table 1:** Demographic and clinical characteristics of the study participants.

	Total Amyloid-PET Positive MCI (N = 126)	No Depression (N = 60)	Depression (N = 66)	*p* Value
Age (years ± SD)	78.02 (6.76)	78.38 (6.41)	77.70 (7.10)	NS
Education (years ± SD)	11.21 (4.72)	11.56 (5.30)	11.09 (4.11)	NS
Sex (M:F)	52:74	27:33	25:41	NS
APOE E4 (yes:no)	65:61	31:29	34:32	NS
SUVR (mean ± SD)	0.757 (0.091)	0.76 (0.094)	0.754 (0.089)	NS
HAM-D (mean ± SD)	10.93 (6.31)	3.783 (1.02)	16.52 (2.53)	NS
CERAD-K Battery (mean ± SD)				
VF	10.61 (3.55)	10.967 (3.48)	10.28 (3.61)	NS
BNT	9.97 (2.57)	9.817 (2.73)	10.1 (2.46)	NS
MMSE	24.65 (2.43)	24.6 (2.3)	24.69 (2.49)	NS
WLM	13.04 (3.26)	13.08 (3.72)	13.00 (2.72)	NS
CP	9.70 (1.44)	9.5 (1.62)	9.88 (1.27)	NS
WLR	2.38 (1.63)	2.267 (1.74)	2.49 (1.55)	NS
WLRc	6.33 (2.52)	6.083 (2.53)	6.54 (2.53)	NS
CR	2.40 (2.09)	2.233 (1.81)	2.53 (2.29)	NS
CERAD total score	53.02 (8.93)	52.65 (9.33)	53.34 (8.51)	NS

APOE: Apolipoprotein E; BNT: 15-Item Boston Naming Test; CERAD-K: The Korean Version of Consortium to Establish A Registry For Alzheimer’s Disease; CDR: Clinical Dementia Rating; CP: Constructional Praxis; CR: Constructional Recall; HAM-D: 17-item Hamilton Depression Rating Scale; MCI: Mild Cognitive Impairment; MMSE: Mini Mental Status Examination; NS: Not Significant, SD: Standard Deviation; VF: PET: Positron Emission Tomography; SUVR: Standardized Uptake Value Ratio for ^18^F-Flutemetamol; Verbal Fluency; WLRc: Word List Recognition; WLM: Word List Memory; WLR, Word List Recall.

## Data Availability

Not applicable.
